# Dynamic behavior of the nucleus pulposus within the intervertebral disc loading: a systematic review and meta-analysis exploring the concept of dynamic disc model

**DOI:** 10.3389/fbioe.2025.1582438

**Published:** 2025-06-06

**Authors:** Jean-Philippe Deneuville, Maxime Billot, Alexandra Cervantes, Sylvain Peterlongo, Martin Meyer, Mezika Kolder, Marie Escande, Mathilde Bourgeois, Adrien Pallot, Romain David, Manuel Roulaud, Amine Ounajim, Mark Laslett, Mathieu Sarracanie, Najat Salameh, Arnaud Germaneau, Philippe Rigoard

**Affiliations:** ^1^ PRISMATICS Lab (Predictive Research in Spine/Neuromodulation Management and Thoracic Innovation/Cardiac Surgery), Poitiers University Hospital, Poitiers, France; ^2^ Association Francophone McKenzie (AFMcK – McKenzie Francophonic Association), Paris, France; ^3^ Institut Pprime UPR 3346, CNRS – Université de Poitiers – ISAE-ENSMA, Poitiers, France; ^4^ Centre Européen d’Enseignement en Rééducation et Réadaptation Fonctionnelle (CEERRF – European Center for Education in Rehabilitation and Functional Rehabilitation), Saint Denis, France; ^5^ Institut d’Ingénierie de la Santé, Université de Picardie Jules Verne, Amiens, France; ^6^ Department of Spine, Pain and Disability Neurosurgery, Poitiers University Hospital, Poitiers, France; ^7^ The Sports Clinic, Christchurch, New Zealand; ^8^ Center for Adaptable MRI Technology, Institute of Medical Sciences, School of Medicine, Medical Sciences and Nutrition, University of Aberdeen, Aberdeen, United Kingdom

**Keywords:** dynamic disc model, intervertebral disc biomechanics, systematic review/meta-analysis, low back pain, directional preference

## Abstract

**Introduction:**

The dynamic disc model (DDM) is a theoretical framework in spine mechanics that theorizes the behavior of the *nucleus pulposus* within the intervertebral disc under various loads. The model predicts displacement of the *nucleus pulposus* away from the bending loads, for example backward displacement of the *nucleus pulposus* with a flexion load. These predictions are regularly used as a theoretical basis for explaining certain disc pathologies, such as disc herniation.

**Methods:**

We screened seven databases (CENTRAL, Embase, MEDLINE, CINAHL, ScienceDirect, Google Scholar, and HAL) up to July 2024, identifying studies through a PRISMA-guided approach that detailed the mechanical transformation (displacement and deformation) of the *nucleus pulposus* under bending load on the intervertebral disc. We conducted a double-blind data extraction and quality assessment of the body of evidence. Finally, we performed a meta-analysis of proportions.

**Results:**

From the 9,269 articles screened, 14 studies were included in the systematic review and meta-analysis. Magnetic Resonance Imaging (MRI) was employed in 92.8% of the studies, revealing four strategies for assessing *nucleus pulposus* transformation. The meta-analysis of asymptomatic subjects’ data demonstrated that the *nucleus pulposus* behavior aligned with dynamic disc model predictions in 85.4% (95% CI = [79.4–91.4]) across spinal regions and bending directions. However, significant heterogeneity and low study quality were noted. Only one study used discography to assess the DDM in a discogenic pain population, identifying discrepancies in *nucleus pulposus* transformation and contrast agent leakage.

**Conclusion:**

Evidence for the dynamic disc model for intact discs is of low strength, whereas very limited evidence challenges the dynamic disc model for fissured discs. New multiparametric MRI studies may help guiding future clinical assessment protocols.

**Systematic Review Registration:**

CRD42022331774.

## Introduction

With an estimated prevalence of approximately 20%, millions of individuals suffer from low back pain (LBP) worldwide (Hoy et al., 2012). LBP represents a tremendous burden for healthcare systems, costing billions of dollars/euros annually (Vos et al., 2016). In some cases, LBP extends beyond a self-limiting painful condition as it translates into disability (Vos et al., 2016) and impacts the quality of life (Ounajim et al., 2021), as well as the psychological (Pincus et al., 2002) and sociological wellbeing of patients (Naiditch et al., 2021b; Naiditch et al., 2021a). Despite a large number of clinical and experimental studies (McKenzie, 1981) exploring LBP over the past few decades, no consensus has been reached regarding the diagnostic process of the LBP population (Maher et al., 2017). In this context, different pathophysiological hypotheses, such as the dynamic disc model (DDM), have been proposed in the literature.

**FIGURE 1:**
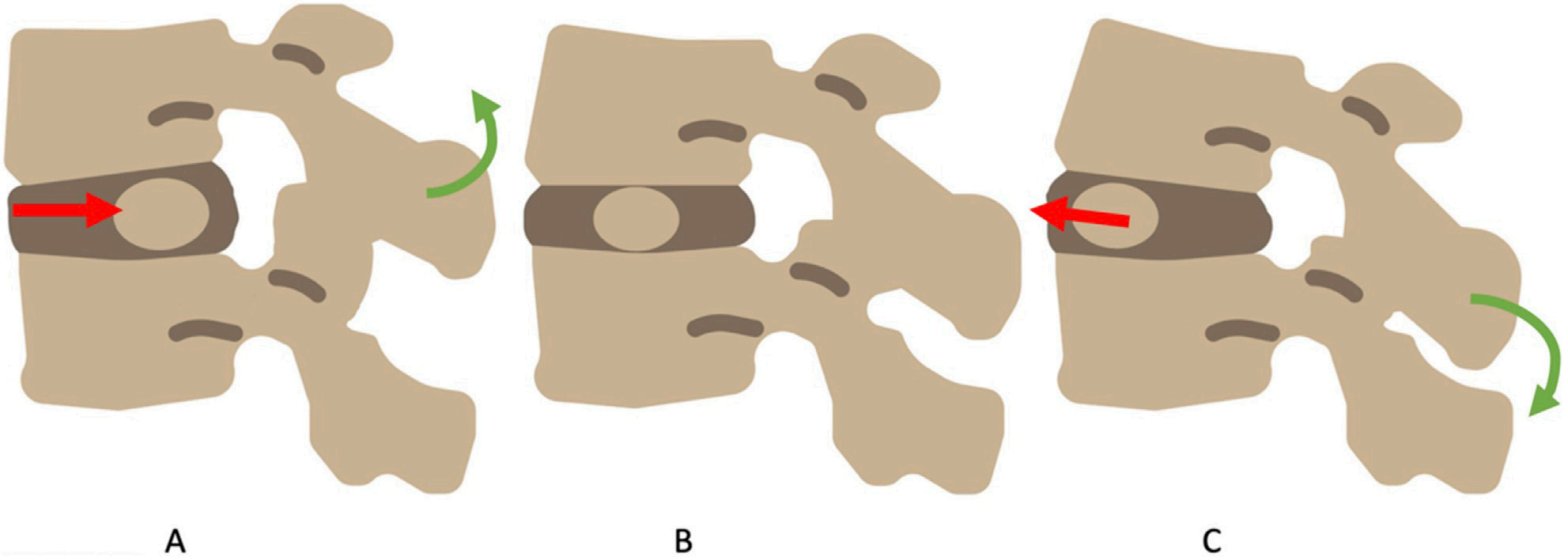
The dynamic disc model, in flexion **(A)** the NP migrates backward, in extension **(C)** the NP migrates forward. In neutral **(B)** the NP is central.

The DDM is based on mechanical transformation, i.e. displacement or strain, of the *nucleus pulposus* (NP) within the intervertebral disc in the opposite direction from pressure applied by bending load (Figure 1). In a healthy disc, the *annulus fibrosus* (AF), containing the NP, would limit the deformation or displacement. Consequently, radial fissures of the *annulus fibrosus* (fissure from the center to the periphery of the AF), may allow the NP to spread into the fissure and stimulate nociceptors localized in the external part of the annulus fibrosus (García-Cosamalón et al., 2010) or surrounding fissure (Lama et al., 2018).

The DDM was used to provide a patho-anatomical explanation for the pain centralization phenomenon (CP) observed in clinical practice (Cyriax, 1950; McKenzie, 1981). CP is defined as the rapid and lasting abolition of distal pain as consequence of spinal bending load, and it occurs in a specific direction, called the directional preference (DP). According to the concept advocated by McKenzie (1981), CP may be the symptomatic expression of migration of the NP material away from the fissure towards the center of the intervertebral disc, while peripherization (opposite to the centralization phenomenon) may reflect the migration of the NP into the fissure.

Fifteen years ago, in a systematic review including 12 studies, Kolber and Hanney (2009) reported that lumbar NP moved opposite to the side of loading in asymptomatic and young subjects for lumbar sagittal bending loads. The authors also reported limited and contradictory results supporting the DDM for older or pathological lumbar discs. In addition, they highlighted a lack of research for the cervical, thoracic, and lumbar spine outside of the sagittal plane. Since then, studies have investigated the NP behavior thanks to new technological advances (Nazari et al., 2012; Fazey et al., 2013; Takasaki, 2015). An updated literature synthesis is now timely for providing new insights into NP mechanical transformation according to the bending load imposed.

This systematic review with meta-analysis aimed to identify and summarize the *in vivo* evidence of *nucleus pulposus* behavior with lumbar, thoracic, and cervical intervertebral disc loading. This review covered both the evidence for intact and for radially fissured discs.

## Methods

The current systematic review and meta-analysis was performed in line with the Preferred Reporting Items for Systematic review and Meta-Analysis (PRISMA) (Page et al., 2021). The protocol for this systematic review was registered on PROSPERO (number CRD42022331774).

### Search strategy

Electronic database MEDLINE, EMBASE, CINAHL, Cochrane Center Register of Controlled Clinical Trials (CENTRAL), ScienceDirect, Google Scholar and Archives Ouvertes HAL were searched until 25 August 2024 with no date limitation no date limitation (see PICO strategy in Table 1). Search results were augmented by direct contact with experts in the field, to identify potential missed references. Search strategies, based on MeSH terms, were developed for MEDLINE, and subsequently adapted for each database. The full search strategy is available in Supplemental Digital Content Section 1.

**TABLE 1:** Summary of the eligibility criteria for the review.

PICO domain	Reviewer choices
Population	Intervertebral human disc studied *in vivo*
Intervention	Radial fissure of the *annulus fibrosus* examined by MRI or discography
Control	Intact *annulus fibrosus*
Outcomes	Direction and length of the deformation/displacement of the NP in terms of the direction of the bending load
Study design	Cross sectional, case-controlled, prospective In case of paucity, case report and case series
Language	French, English, Spanish and Portuguese

### Study selection

All results were imported to a citation manager (Zotero 6.0.26) and duplicate entries were merged before importing the final list into the Rayyan website for the review process. Each article was screened for eligibility twice, independently, and blindly, by two authors, first by title/abstract, then by full article reading. In case of disagreement, a third reviewer arbitrated the final decision for inclusion/exclusion. The list of excluded articles and the reasons for their exclusion are available in Supplemental Digital Content Section 2 (starting from the full-text review stage).

### Eligibility criteria

Every study that assessed and reported the mechanical transformation of NP during or after bending loads was included. We considered cross-sectional, case-controlled, or prospective studies. We considered human intervertebral discs studying *in vivo* conditions within asymptomatic or low back pain populations. Low back pain patients must have been screened for discogenic pain or radial fissure prior to the DDM assessment. Full scientific articles written in English, French, Spanish or Portuguese were considered.

### Intervention/exposition

We considered intervertebral painful disc with radial fissures according to the North American Spine Society (NASS) classification. NASS defined radial fissure as “[…] disruption of annular fibers extending from the *nucleus pulposus* outward toward the periphery of the *annulus fibrosus*, usually in the cranial-caudad (vertical) plane, although, at times, with axial horizontal (transverse) components […]”(Fardon et al., 2014).

### Data extraction

Data of interest were extracted from eligible articles twice, independently, and blindly. Information from considered studies included authors, year of publication, country, funding, participants characteristics (age, gender, symptomatic/asymptomatic status), MRI characteristics (magnetization, slice thickness, field of view, matrix dimensions, echo and repetition time), image acquisition plan, type of bending load, method of image analysis. Disagreements were resolved through discussion or, if necessary, by a third independent reviewer.

### Risk of bias and reporting bias assessment

Risk of bias of each eligible article was rated twice, independently, and blindly, using the Newcastle-Ottawa scale (NOS). Disagreements were resolved through discussion or, if necessary, by a third reviewer. The scale evaluates the following domains: representativeness of sample, sample size justification, non-respondents, ascertainment of the exposure, comparability, assessment of outcome.

For the meta-analysis, reporting bias was assessed with the LFK index and Doi plot (Furuya-Kanamori et al., 2018). While Egger index and funnel plot (Higgins and Collaboration, 2020) are commonly used, recent evidence demonstrated the superiority of LFK index and Doi plot, especially for proportion meta-analysis (Hunter et al., 2014; Furuya-Kanamori et al., 2018; Cheema et al., 2022).

### Certainty assessment

To assess the certainty of evidence, the Grading of Recommendations Assessment, Development, and Evaluation (GRADE) was used (Guyatt et al., 2008). Certainty of evidence was rated as high, moderate, low, or very low according to risk of bias, heterogeneity, and methodological quality of the study.

### Quantitative synthesis method

Two proportion meta-analyses were performed, one to assess intact disc among pain-free subjects and a second one to assess fissured disc among discogenic pain population. We used a proportion meta-analysis to determine the proportion of experimental observation in which the disc matches with the DDM, *in vivo*. We defined a DDM match as the NP moving/deforming away from the direction of the bending load, e.g., NP moving/deforming backward when flexion bending loads were imposed to the disc. We define an experimental observation as an observation in which the disc is bent, e.g., if a study assesses 10 discs in flexion and extension with respect to neutral position, the study includes a total of 20 observations. We conducted proportion meta-analysis for intact and fissured discs independently.

We used a random-effect model for gathering data, considering the potential high variability between studies. Heterogeneity was quantified with I^2^ statistic and the Cochrane’s Q test. We evaluated heterogeneity graphically using Forest plots. In case of insufficient information report, we contacted the authors of the study to request data sharing. If the requested information was not available, the study was excluded from the meta-analysis.

Analyses were performed using R language (version: R 4.2.2. GUI 1.79; interface: Rstudio 2022.07.2; package: *meta, metafor, metasens and dosresmeta*; operating system: MacOS Ventura 13.2).

## Results

### Study selection

**FIGURE 2:**
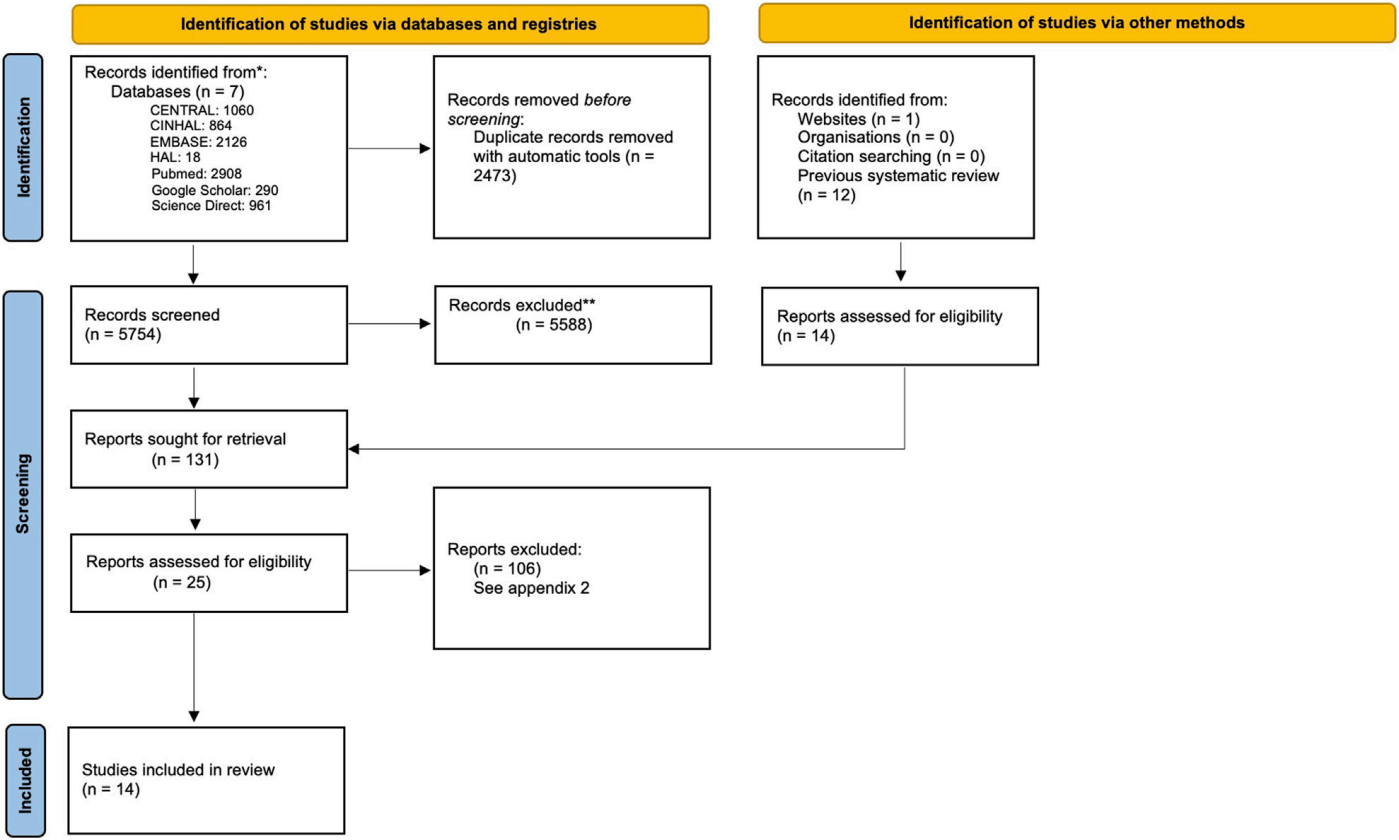
PRISMA flow chart of the review.

The PRISMA flow chart detailing the screening process is presented in Figure 2. The initial database research identified 9,269 articles. After removing 3,164 duplicates, 6,105 articles were selected for abstract and title assessment, and 25 studies were analyzed as full-text publications. After full-text screening, we included 14 articles in the final systematic review and 10 in the meta-analysis. The reliability of the full inclusion process was good (Cohen’s Kappa = 0.85 CI95% = [0.73–0.96] and %_agreement_ = 0.998) (Landis and Koch, 1977).

Eight studies initially identified in the systematic review by Kolber and Hanney, (2009) were not included in the present review.

Four of these studies were cadaveric studies (Shah et al., 1978; Gill et al., 1987; Krag et al., 1987; Seroussi et al., 1989). The others evaluated the deformation of the annular wall, via MRI, in subjects with or without low back pain, during trunk movements (Zamani et al., 1998; Weishaupt et al., 2000; Fredericson et al., 2001; Parent et al., 2006). Despite the value of annular wall deformation data for understanding the mechanics of the intervertebral disc in a broad sense, they are not direct observation of the NP transformations. Therefore, they did not assess the DDM, hence the decision to exclude these articles. In this regard, we identified several studies conducted after the review by Kolber and Hanney (2009), assessing annular deformation (Zou et al., 2008; 2009), that were not included. Lastly, one additional study was excluded although the methods described were very close to our inclusion criteria (Abdollah et al., 2018). In this study, the authors identified the position of the NP via MRI in subjects with low back pain before and after repeated extension movements. However, these authors considered a population of low back pain patients without first identifying those suffering from discogenic pain or comparing target disc(s) with intact and pain-free ones.

### Study characteristics

A comprehensive review of study characteristics is reported in Table 2. The earliest study dated from 1988 and latest from 2019.

**TABLE 2:** Study characteristics.

Studies	Funding	Country	Design	*Ex/in vivo*
Schnebel et al. (1988)	Not reported	United States	Cross sectional	*In vivo*
Beattie et al. (1994)	Supported by a grant from the state of New Mexico to the Center for Non-Invasive Diagnosis, School of Medicine, University of New Mexico (87,131)	United States	Cross sectional	*In vivo*
Fennell et al. (1996)	Not reported	United Kingdom	Case series	*In vivo*
Brault et al. (1997)	Supported by a Rose McNutt Osteopathic Research Fellowship and AOA-92–01–348	United States	Cross sectional	*In vivo*
Edmonston et al. (2000)	Not reported	Australia	Cross sectional	*In vivo*
Périé et al. (2001)	Authors acknowledge the Société LAGARRIGUE for their financial support	France	Cross sectional	*In vivo*
Fazey et al. (2006)	Not reported	Australia	Cross sectional	*In vivo*
Alexander et al. (2007)	Not reported	United Kingdom	Cross sectional	*In vivo*
Fazey et al. (2010)	Not reported	Australia/Japan	Cross sectional	*In vivo*
Nazari et al. (2012)	Not reported	United Kingdom	Cross sectional	*In vivo*
Fazey et al. (2013)	Not reported	Australia	Cross sectional	*In vivo*
Takasaki (2015)	Not reported	Australia/Japan	Cross sectional	*In vivo*
Kim et al. (2017)	This work was supported by Seoul National University Hospital (Grant No. 0320110030) and Korea Foundation for the Advancement of Science and Creativity (KOFAC) grant funded by Korean Ministry of Education, Science and Technology (MEST)	Corea	Cross sectional	*In vivo*
Elmaazi et al. (2019)	The study was financially supported by Manchester Metropolitan University and there is no financial benefit for the authors	United Kingdom	Cross sectional	*In vivo*

Thirteen *in vivo* studies (92.8%) evaluated the dynamic behavior of the NP in young adults with intact discs (ranging in age from 22.4 to 37 years) (Beattie et al., 1994; Fennell et al., 1996; Brault et al., 1997; Edmondston et al., 2000; Périé et al., 2003; Fazey et al., 2006; 2010; 2013; Alexander et al., 2007; Nazari et al., 2012; Takasaki, 2015; Kim et al., 2017), while one study assessed the dynamic behavior of the NP in chronic low back pain population (Schnebel et al., 1988), including subjects eligible for awake discography at three levels (Schnebel et al., 1988).

Thirteen studies (92.8%) used T2-weighted MR images (T2_w_-MRI) for assessing the dynamic behavior of the NP (Beattie et al., 1994; Fennell et al., 1996; Brault et al., 1997; Edmondston et al., 2000; Périé et al., 2003; Fazey et al., 2006; 2010; 2013; Alexander et al., 2007; Takasaki H et al., 2010; Nazari et al., 2012; Takasaki, 2015; Kim et al., 2017; Elmaazi et al., 2019), while one used discography (Schnebel et al., 1988).

Two studies focused on the NP behavior of cervical discs in the sagittal plane (Kim et al., 2017; Elmaazi et al., 2019)when a sagittal bending load was applied (flexion/extension), while six others focused on lumbar discs (Fennell et al., 1996; Edmondston et al., 2000; Fazey et al., 2006; 2010; 2013; Alexander et al., 2007). Among these six studies, two also assessed the effect of axial rotation (axial plane) on NP transformation in the frontal plane (Fazey et al., 2006; 2013). Additionally, two different studies assessed the effect of frontal plane bending load (side glide and side bending) on the transformation of the lumbar NP in the frontal plane (Périé et al., 2003; Takasaki, 2015).

### Risk of bias

Methodological quality assessment of the retained studies is reported in Table 3. The overall quality is low to fair, and risk of bias is high (score on the NOS ranging from 3 to 7). Two (14.2%) (Alexander et al., 2007; Fazey et al., 2010; Nazari et al., 2012) scored above six out of 10 and one scored 7 (7.1%) (Alexander et al., 2007). In all studies, we did not find any attempt at *a priori* sample size justification (Gill et al., 1987; Schnebel et al., 1988; Beattie et al., 1994; Brault et al., 1997; Edmondston et al., 2000; Périé et al., 2003; Fazey et al., 2006; Fazey et al., 2010; Fazey et al., 2013; Alexander et al., 2007; Nazari et al., 2012; Takasaki, 2015; Kim et al., 2017; Elmaazi et al., 2019). In all of the 13 studies including asymptomatic subjects (92.8%), only young people with no sign of disc degeneration or pathologies were included. The 10 (71.4%) lower ranked studies did not properly describe methods used to assess the main outcome (e.g., blind analysis) (Gill et al., 1987; Schnebel et al., 1988; Beattie et al., 1994; Brault et al., 1997; Edmondston et al., 2000; Périé et al., 2003; Fazey et al., 2006; 2013; Takasaki, 2015; Kim et al., 2017). Nearly all the studies used methods which ascertain the direction of movement imposed to the vertebral unit properly (either with MRI or discography). Finally, 11 studies (78.6%) performed statistical analysis (with confidence intervals) (Beattie et al., 1994; Brault et al., 1997; Edmondston et al., 2000; Périé et al., 2003; Alexander et al., 2007; Fazey et al., 2010; 2013; Nazari et al., 2012; Takasaki, 2015; Kim et al., 2017; Elmaazi et al., 2019).

**TABLE 3:** Risk of bias assessment, Newcastle-Ottawa rating scale.

Studies	Representativeness of sample	Sample size	Non-respondents	Ascertainment of the exposure	Comparability	Assessment of outcome	Statistical test	Total
Schnebel et al. (1988)	—	—	—	**	—*	—	—	3
Beattie et al. (1994)	-	—	—	**	—*	—	*	4
Fennell et al. (1996)	NA	NA	NA	NA	NA	NA	NA	NA
Brault et al. (1997)	—	—	—	**	—*	—	*	4
Edmonston et al. (2000)	—	—	—	**	—*	—	*	4
Périé et al. (2001)	—	—	—	**	—*	—	*	4
Fazey et al. (2006)	—	—	—	**	—*	—	—	3
Alexander et al. (2007)	—	—	—	**	**	**	*	7
Fazey et al. (2010)	—	—	—	**	—*	**	*	6
Nazari et al. (2012)	—	—	—	**	—*	**	*	6
Fazey et al. (2013)	—	—	—	**	—*	—	*	4
Takasaki (2015)	—	—	—	**	—*	—	*	4
Kim et al. (2017)	—	—	—	**	—*	—	*	4
Elmaazi et al. (2019)	—	—	—	**	—*	—*	*	5

* : item with one point and one point validated.

** : item with two points and both validated.

—* : item with two points but only one validated.

— : no point validated.

**FIGURE 3:**
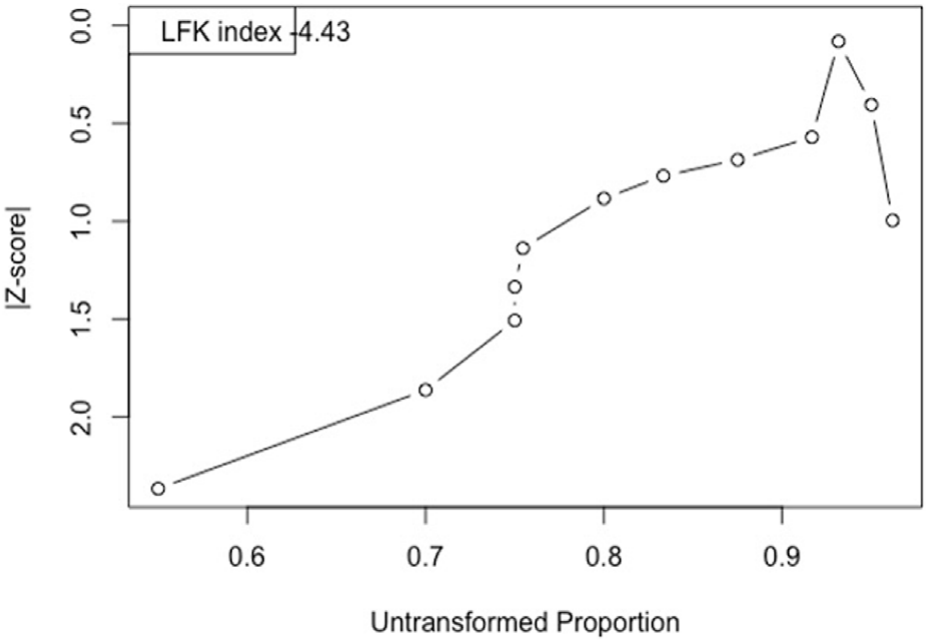
Doi plot and LFK index for the meta-analysis.

Reported bias assessment of the meta-analysis (meta-proportion for objective 1, Figure 3) is high with a LFK index = −4.43 and a left skewed asymmetric Doi plot.

## Results of syntheses

### Narrative synthesis of the dynamic behavior of the NP assessment

We identify two image acquisition methods (T2_w_-MRI and discography procedures) and four image analysis methods (distance boundaries, peak pixel intensity, pixel intensity profilometry, centroid method).

Regarding image acquisition, 13 studies utilized MRI (Beattie et al., 1994; Fennell et al., 1996; Brault et al., 1997; Edmondston et al., 2000; Périé et al., 2003; Fazey et al., 2006; Fazey et al., 2010; Fazey et al., 2013; Alexander et al., 2007; Takasaki H et al., 2010; Nazari et al., 2012; Takasaki, 2015; Kim et al., 2017; Elmaazi et al., 2019), with significant heterogeneity in the acquisition methods.

• Magnetic field ranged from 0.2 to 3 T.
• Slice thicknesses ranged from 4 to 8 mm.
• Field of view ranged from 18 to 35 cm.
• Echo time ranged from 16 to 120 milliseconds.
• Repetition time ranged from 312 to 5,160 milliseconds (see Table 4).
• No study reported in-plane pixel sizes.

**TABLE 4:** Data extracted for study of interest.

Studies	Sample size	Age^1^ years	Gender^2^	Spine section studied	Type of population	Image acquisition modalities	Image acquisition plan	Type of bending load	Method of image analysis
Schnebel et al. (1988)	35	37 (NA)	NA (NA)	Lumbar (L3 to S1)	Chronic low back pain subject with • Symptomatic discs • Asymptomatic disc	Discography with X-ray to assess contrast agent diffusion	Sagittal	Flexion Extension	Distance boundaries
Beattie et al. (1994)	20	23.8 (sd: 2.6)	20 (100%)	Lumbar (L3 to S1)	Asymptomatic subjects	T2_w_ MRI Magnetic field: 1.5 T Slice thicknesses: 5 mm Field of view: 18 cm Matrix: 256 Echo time: 30/80 ms Repetition time: 2000 ms In-plane pixel sizes: 0.703 × 0.703 mm	Sagittal	Flexion Extension	Distance boundaries
Fennell et al. (1996)	3	29.7 (sd: 14.6)	1 (33.3%)	Lumbar (L1 to L5)	Asymptomatic subjects	T2_w_ MRI Magnetic field: 1.5 T Slice thicknesses: 4 mm Field of view: NR^3^ Matrix: NR Echo time: NR Repetition time: 3,000 ms In-plane pixel sizes: NR	Sagittal	Flexion Extension	Distance boundaries
Brault et al. (1997)	10	NA (r: 21–38)	0 (0%)	Lumbar (L1 to S1)	Asymptomatic subjects	T2_w_ MRI Magnetic field: 1.5 T Slice thicknesses: 4 mm Field of view: 28 cm Matrix: 256 × 256 Echo time: 17/32 ms Repetition time: 2000/3,000 ms In-plane pixel sizes: 1.094 × 1.094 mm	Sagittal	Flexion Extension	Peak pixel intensity
Edmonston et al. (2000)	10	30 (sd: 5.8)	6 (60%)	Lumbar (L1 to S1)	Asymptomatic subjects	T2_w_ MRI Magnetic field: 1 T Slice thicknesses: 4 mm Field of view: 30 cm Matrix: 512 × 512 Echo time: 30 ms Repetition time: 3,000 ms In-plane pixel sizes: 0.586 × 0.586 mm	Sagittal	Flexion Extension	Peak pixel intensity
Périé et al. (2001)	14	NA (NA)	NA (NA)	Thoraco-lumabar	Asymptomatic subjects	T2_w_ MRI Magnetic field: 1.5 T Slice thicknesses: NR Field of view: NR Matrix: NR Echo time: NR Repetition time: NR In-plane pixel sizes: NR	Frontal	Side bending	Centroid
Fazey et al. (2006)	3	27 (NA)	3 (100%)	Lumbar (L1-L2 and L4-L5)	Asymptomatic subjects	T2_w_ MRI Magnetic field: 1 T Slice thicknesses: NR Field of view: 35 cm Matrix: 256 × 256 Echo time: 113 ms Repetition time: 4,300 ms In-plane pixel sizes: 1.367 × 1.367 mm	Axial	Flexion Extension Left rotation	Pixel intensity profilometry
Alexander et al. (2007)	11	36 (sd: 9)	7 (63.6%)	Lumbar (L1 to S1)	Asymptomatic subjects	T2_w_ MRI Magnetic field: 0.6T Slice thicknesses: 4.5 mm Field of view: 30 cm Matrix: 180 × 256 Echo time: 120 ms Repetition time: 3,484 ms In-plane pixel sizes: 1.667 × 1.172 mm	Sagittal	Flexion Extension	Peak pixel intensity
Fazey et al. (2010)	21	24.8 (r: 20–34)	11 (52.4%)	Lumbar (L1 to S1)	Asymptomatic subjects	T2_w_ MRI Magnetic field: 0.2 T Slice thicknesses: 8 mm Field of view: 26 cm Matrix: NR Echo time: 120 ms Repetition time: 3,120 ms In-plane pixel sizes: NR	Axial	Side bending	Pixel intensity profilometry
Nazari et al. (2012)	25	26.8 (sd: 5.6)	0 (0%)	Lumbar (L3 to S1)	Asymptomatic subjects	T2_w_ MRI Magnetic field: 0.6 T Slice thicknesses: 4.5 mm Field of view: 30 cm Matrix: 256 × 256 Echo time: 100 ms Repetition time: 2,376 ms In-plane pixel sizes: 1.172 × 1.172 mm	Sagittal	Flexion Extension	Distance boundaries
Fazey et al. (2013)	10	29 (r: 24–34)	5 (50%)	Lumbar (L1-L2 and L4-L5)	Asymptomatic subjects	T2_w_ MRI Magnetic field: 1.5 T Slice thicknesses: NR Field of view: 21 cm Matrix: 384 × 384 Echo time: 102 ms Repetition time: 5,160 ms In-plane pixel sizes: 0.547 × 0.547 mm	Axial	Extension Flexion Left rotation	Pixel intensity profilometry
Takasaki (2015)	20	24.8 (sd: 4)	10 (50%)	Lumbar (L1 to S1)	Asymptomatic subjects	T2_w_ MRI Magnetic field: 0.2 T Slice thicknesses: 8 mm Field of view: 26 cm Matrix: NR Echo time: 120 ms Repetition time: 312 ms In-plane pixel sizes: NR	Axial	Side bending Side gliding	Pixel intensity profilometry
Kim et al. (2017)	10	22.4 (sd: 1.6) (r: 19–30)	0 (0%)	Cervical (C3 to C7)	Asymptomatic subjects	T2_w_ MRI Magnetic field: 3T Slice thicknesses: 3 mm Field of view: 28 cm Matrix: 448 × 448 Echo time: 104 ms Repetition time: 4,000 ms In-plane pixel sizes: 0.625 × 0.625 mm	Sagittal	Extension	Distance boundaries
Elmaazi et al. (2019)	25	33.7 (sd: 9.1) (r: 21–49)	15 (60%)	Cervical (C5 to C7)	Asymptomatic subjects	T2_w_ MRI Magnetic field: 0.2 T Slice thicknesses: 4 mm Field of view: 26 cm Matrix: 256 × 256 Echo time: 24 Repetition time: 650 In-plane pixel sizes: 1.016 × 1.016 mm	Sagittal	Flexion Extension	Distance boundaries

^1^ Expressed as a mean.

^2^ Percentage of women.

Only one study utilized discography to obtain the images (Schnebel et al., 1988). The authors described a lateral approach for the contrast agent injection and used X-ray from a lateral plane to observe the diffusion of the contrast agent.

Regarding the four image analysis methods: (i) Five studies (Beattie et al., 1994; Fennell et al., 1996; Nazari et al., 2012; Kim et al., 2017; Elmaazi et al., 2019) utilizing MRI and the one utilizing discography (Schnebel et al., 1988) used distance boundaries. The latter consist in calculating the distance from the margin of the NP to the margin of disc and two of those studies reported (Kim et al., 2017; Elmaazi et al., 2019) appropriate reliability indicators with ICC ranging from 0.77 to 0.94 and SEM from 0.26 to 0.37 mm. (ii) Three studies (Brault et al., 1997; Edmondston et al., 2000; Alexander et al., 2007) used peak pixel intensity consisting in identifying the position of the maximum pixel intensity of the NP on a line which bisects the anterior and posterior limits of the intervertebral disc on a T2_w_ image. The distance from the peak pixel intensity to the anterior limit of the disc was measured. Then the distance was converted from mm to % of the antero-posterior diameter of the intervertebral disc. Two studies reported ICC ranging from 0.71 to 0.97, and one reported SEM of 4.3% (Edmondston et al., 2000; Alexander et al., 2007). (iii) Four studies (Fazey et al., 2006; Fazey et al., 2010; Fazey et al., 2013; Takasaki, 2015) used pixel intensity profilometry, which consists of extracting grayscale intensity values from T2-weighted MRI images across the full diameter of the intervertebral disc. Specifically, the intensity values were sampled along 3 to 55 image matrix lines (i.e., rows or columns of the MRI image matrix) that passed through the nucleus pulposus, spanning from one boundary of the disc to the other (e.g., right to left). Each line was rescaled to a 100-point normalized axis to account for variations in disc size. The intensity values were then plotted as a function of this normalized position, generating a pixel intensity profile. Two main parameters were derived from this curve: (i) the distance between the point of maximum intensity and the nearest disc boundary, and (ii) the centroid of the area under the curve, defined as the point dividing the total area under the intensity curve into two equal halves. One study (Takasaki, 2015) reported an ICC >0.8, the others do not report reliability measurement. (iv) One study (Périé et al., 2003) used the centroid method consisting in determining the centroid by segmenting the NP and the two adjacent vertebrae. After computing the centroids of each vertebra, a midpoint was established between them. The distance from this midpoint to the NP’s centroid was then measured. The study (Périé et al., 2003) did not report ICC or SEM values.

All the MRI studies assessed intact discs, with a NP behavior consistent with the prediction of the DDM. In the discography study, the authors assessed patients with both normal and abnormal discs. Abnormal discs were those in which the injection reproduced the patient’s pain, allowing for a classification as discogenic pain, but the authors did not report more information regarding the morphology of the disc (presence of a fissure, size, orientation). From our results, it is the only study assessing the behavior of the NP in a discogenic pain population. The contrast agent did not move as predicted by the DDM for abnormal discs, whereas it did for the normal disc. They also reported an opposite behavior with the L5-S1 abnormal disc, where the contrast agent was seen to migrate posteriorly with an extension load.

### Quantitative synthesis

From the 10 analyzed studies, the global proportion of NP behaving as predicted by the DDM was 85.4% (CI95% = [79.4–91.4]). The NP behavior as predicted by the DDM was 86% (CI95% = [76.8–95.2]) for deformation of the NP in the sagittal plane following lumbar sagittal bending load (flexion/extension), 83.2% (95CI% [73.27–93.2]) for deformation of the NP in the sagittal plane following cervical sagittal bending load (flexion/extension), and 90.4% (95CI% = [81.9–98.9]) for deformation of the NP in the frontal plane following lumbar frontal bending load (side bending or side gliding).

Regarding lumbar axial rotation, the interpretation of NP deformation remains uncertain due to a lack of clarity in the experimental setups described in the two original studies. In particular, one study reported inducing “left trunk rotation” by placing a foam wedge under the left hemipelvis, but without specifying whether the shoulders were constrained or allowed to follow the rotation. As a result, the actual orientation of trunk rotation—clockwise or counterclockwise from a cranio-caudal perspective—cannot be determined with certainty. Because this information is essential to assess whether the nucleus pulposus moved in the same direction as the rotation or in the opposite direction (as predicted by the DDM), these data could not be reliably interpreted. For this reason, the results from these two studies were excluded from the meta-analysis.

**FIGURE 4:**
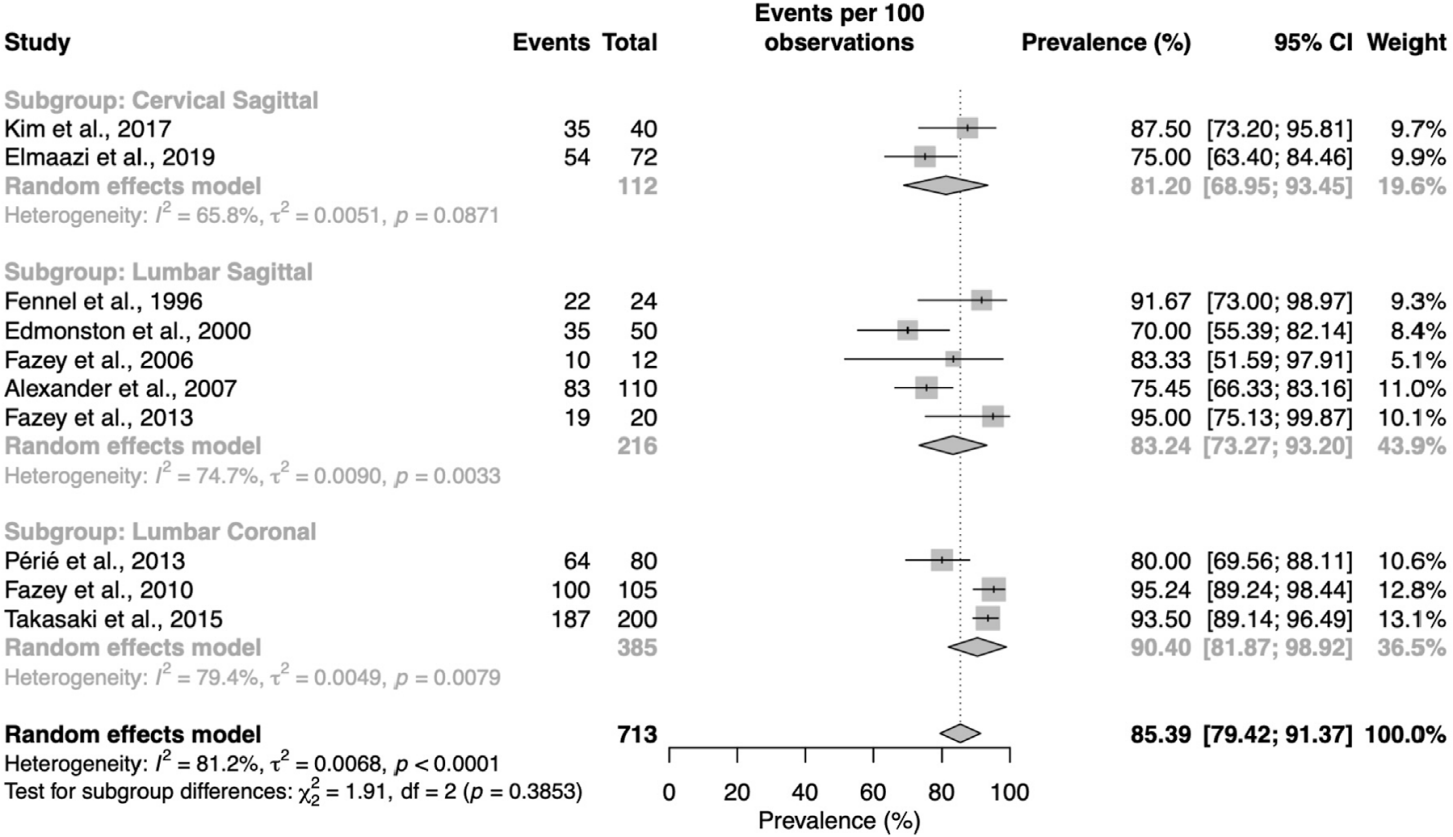
Proportion meta-analysis for intact discs and doi plot. The proportion is the total cases of interest (column Number) representing the number of experimental observations in which the DDM is confirmed. The size of the sample (column Total) represents the total number of observation), e.g., if a study assesses 10 discs in flexion and extension, we have a sample size of 20 observations.

Heterogeneity was high with I^2^ = 81.2% (Figure 4).

## Discussion

Our systematic review included 14 studies. It showed a consistent behavior of the NP across spinal levels and plans of movement among young and asymptomatic subjects with no pathological disc. We identified two different imaging modalities and four image analysis methods to characterize the NP behavior. Our meta-analysis included 10 studies. It showed that the NP behavior supported the dynamic disc model (DDM) in 85.4% of the observations, with 83.2% in the sagittal plane following lumbar sagittal bending load, 81.2% in the sagittal plane following cervical sagittal bending load, and 90.4% in the frontal plane following lumbar frontal bending load. Studies showed low quality evidence for intact discs and high heterogeneity (I^2^ = 81.2%) (Shah et al., 1978; Beattie et al., 1994; Fennell et al., 1996; Brault et al., 1997; Edmondston et al., 2000; Périé et al., 2003; Fazey et al., 2006; 2010; 2013; Alexander et al., 2007; Nazari et al., 2012; Takasaki, 2015; Kim et al., 2017; Elmaazi et al., 2019).

### The dynamic disc model for intact and fissured discs

Our results for lumbar intact intervertebral discs subjected to flexion/extension tend to align with those of the previous systematic review (Kolber and Hanney, 2009). In addition to this trend, we conducted a meta-analysis and determined that 85.4% [79.4–91.4] of the observations were coherent with the prediction of the Disc Displacement Model (DDM). Moreover, in contrast to the previous review, we identified results for the lumbar spine in other planes of movement, with side bending/gliding (90.4% [79.4–98.9]), as well as for the cervical spine in the sagittal plane (81.2% [68.9–93.4]). These meta-analyses and results for other planes of movement and spine regions allow a more precise understanding of the behavior of the nucleus pulposus (NP) when the intervertebral disc is subjected to loads.

Although the DDM provides clear predictions for NP behavior under sagittal and frontal bending loads, its applicability to rotational movements remains uncertain. In this review, two studies investigated the deformation of the NP during axial trunk rotation (Fazey et al., 2006; Fazey et al., 2013). However, due to methodological ambiguities, it is not possible to draw meaningful conclusions from their results. In particular, these two studies described inducing “left trunk rotation” by placing a foam wedge under the left hemipelvis, but did not specify whether the shoulders were fixed or allowed to follow the pelvic movement. This omission makes it impossible to determine the actual orientation of trunk rotation (i.e., clockwise or counterclockwise from a cranio-caudal perspective), which is a critical parameter for interpreting the direction of NP displacement in relation to the DDM.

Furthermore, both studies lacked sufficient detail on the kinematic conditions to distinguish whether the applied loads represented pure axial rotation or were combined with side bending or gliding. As interpretation of NP behavior in the DDM relies on well-defined loading directions, this lack of clarity prevents any reliable comparison. For these reasons, the data from these two studies were excluded from the meta-analysis, and no conclusions can be drawn regarding the effect of axial rotation on NP displacement within the framework of the DDM.

Although promising in most of the different bending planes, the heterogeneity of the results compels caution when interpreting them. Nazari et al. (2012) provided another interpretation of the NP behavior, suggesting an elongation of the *nucleus pulposus* with flexion. They argued that elongation of the NP with flexion shortens the distance between the posterior wall of the NP and the posterior wall of the *annulus fibrosus*, inducing a perceptual impression of backward displacement. However, the distance between the anterior wall of the annulus and the nucleus also shortens, demonstrating a global elongation of the NP and not a backward displacement. The opposite phenomenon (contraction of the NP) is observed with extension loads. Whereas the data presented by Nazari et al. (2012) support elongation/contraction (strain) of the NP hypothesis, no extensive literature was available for supporting this. The authors suggested that the illusion of movement measured in the other studies results from the measurement methods limited to only a part of the NP and not the whole NP. Rather than viewing Nazari et al.'s hypothesis as entirely opposing the DDM, we could suggest that both mechanisms (displacement and strain) may coexist. Consequently, the current results suggesting nucleus pulposus displacement could be overestimated due to the concurrent phenomenon of strain. The contraction/elongation phenomenon of the NP, combined with displacement of the NP, makes the transformation of NP following bending loads more complex than expected. This hypothesis should be considered in future research.

### The dynamic disc model for fissured discs

One study evaluated the DDM in the context of discogenic pain using provocative discography (Schnebel et al., 1988). In this study, the authors analyzed the behavior of the contrast agent injected into the disc during flexion and extension movements. The contrast agent injected into discs identified as abnormal through discography exhibited erratic and unpredictable behavior. In the case of L5/S1 levels, this behavior was even contrary to the expected behavior. However, several limitations must be considered. Firstly, Schnebel et al. (1988) classified discs as normal or abnormal but did not clearly specify the presence of a radial fissure or its orientation. Radial fissures can be oriented in any direction within the disc (Yu et al., 1988; Saifuddin et al., 1998; Bogduk et al., 2013). To properly test the DDM, the bending load should be oriented in the direction of the fissure. If this condition is not met, erratic results could be obtained. Secondly, the dimensions of the fissure can sufficiently alter the disc to the point of rendering the DDM inoperative. In this scenario, the DDM may not be applicable in all cases of radial fissures but only in those where the fissure exists in certain dimensions. Thirdly, as these data suggested, the disc could behave differently depending on the level of the spine under study, especially noticeable in L5-S1. Lastly, the differences in mechanical properties (Iatridis et al., 1996) between the contrast agent (liquid) and the NP (viscoelastic solid) might cause the unpredictable behavior. This finding aligns with an *ex vivo* study (not included in this review), suggesting that the DDM is invalid in the context of a fissured disc (Gill et al., 1987). However, no firm conclusion on the invalidity of the DDM in a context of fissured disc could be made due to the paucity of published work in the field as well as the methodological issues raised previously. Future research is necessary.

### Methodological considerations for assessing DDM

Two imaging modalities were identified for the evaluation of DDM. On the one hand, discography was used in one study retained in this work and several excluded studies (*ex vivo*) (Shah et al., 1978; Gill et al., 1987; Schnebel et al., 1988). This method involves the injection of a contrast agent into the patient’s disc. The liquid induces mechanical distension of the inner part of the disc, replicating the primary consultation pain if the disc is to be considered a source of nociception. In healthy discs, participants reported discomfort or atypical pain only. Using X-ray or CT scan, diffusion of the contrast agent within the disc could be observed (Bogduk, 2013), as a component of the criteria for establishing the diagnosis of discogenic pain. To our knowledge, this approach is the only one that provides visualization of the shape, size, and orientation of disc’s radial fissure *in vivo*. Yet, X-ray/CT scan-based discography presents two major drawbacks in addition to being ionizing. First, discography is invasive and seem to accelerate the degeneration of the disc (Carragee et al., 2009), which could limit its use in future research. Secondly, discography is limited as it provides visualization of the contrast agent only, and not the NP. Therefore, inferences from studies using discography are based on the behavior of the contrast agent rather than that of the NP.

The second approach relies on T2-weighted MR images (T2_w_) and does not use contrast medium injection, making it not only non-ionizing but also non-invasive as opposed to discography. Additionally, T2_w_ MRI allows for direct visualization of the NP, facilitating direct measurement of mechanical transformations. However, T2_w_ MRI suffers from different limitations. To our knowledge, direct visualization of the radial fissure in the *annulus fibrosus in vivo* using MRI has never been reported using T2-weighted images. This limitation does not cause concerns regarding DDM research in healthy discs, but leads to major issues when evaluating DDM in the context of a fissured disc. Some authors describe changes in the signal within the AF, called a High-Intensity Zone (HIZ) (Aprill and Bogduk, 1992), that might be wrongly attributed to radial fissure visualization, although it seems to reflect the granulation tissue infiltrating the radial fissure instead (Ross et al., 1990). Finally, contrast in T2_w_ MRI is influenced by the age and tissue degeneration, illustrated by decreased NP signal intensity (Pfirrmann et al., 2001). Therefore, in aging populations, differentiating between the NP and AF is less evident with T2_w_ MRI, making image analysis challenging.

To date, despite their usability, both discography and T2_w_ MRI provide limited information about the complexity of characterizing the NP and its mechanical response to loading in patients presenting with potentially fissured discs.

### Future research perspectives and recommendations

Recent imaging developments combine different MRI methods, including quantitative techniques like diffusion (Beattie PF et al., 2009), relaxometry (Deneuville et al., 2021) and spectroscopy (Gornet et al., 2019), to enable a multiparametric approach (Stabile et al., 2020). Relaxometry computes pixel-wise maps of the absolute T1 and T2 relaxation times of the targeted structures, while T2_w_ MRI sequences provide images in shades of gray which contrast highly depending on the acquisition parameters and MRI hardware. Hence, quantitative T1 and T2 relaxometry mapping provides more objective and consistent imaging compared to conventional T2-weighted sequences (Deoni, 2010). In a proof-of-concept study on cadaveric ovine samples, Deneuville et al. (2021), employed quantitative relaxometry mapping to detect radial fissures in the AF and to monitor NP migration under bending loads, both inside and outside the fissure. Diffusion MRI captures the diffusion of water molecules in tissues. Considering the NP’s high-water content, diffusion MRI emerges as a pertinent choice. Beattie et al. (2009) leveraged this feature and measured variations in the NP’s apparent diffusion coefficient following manual therapy loading procedures. Magnetic resonance spectroscopy (MRS) is sensitive to chemical shift differences between molecules and informs on their concentration levels in tissues. Gornet et al. (2019) demonstrated the capability of MRS to distinguish between painful and non-painful discs. Compared to discography, MRS was able to classify 206 painful/non-painful discs with an average 80% sensitivity and 86% in specificity, which increased to 91% and 93% respectively in non-herniated discs. By combining T2_w_ MRI and a deep learning algorithm, Waldenberg et al. (2022) were able to accurately identify radial fissures (100% sensitivity and 97% specificity) compared to discography. However, despite this very good diagnostic performance, their method did not allow for the direct visualization of the fissure itself—as regards its orientation, length, or precise anatomical extent. This recent advance in MRI data processing opens new avenues for radial fissure detection and characterization. Beyond basic research, visualizing radial fissures in the AF, pinpointing NP positioning, and correlating with symptoms and symptom changes could be a new clinical tool. Multiparametric MRI might supersede invasive discography, potentially guiding clinical decisions for surgeons, physiotherapists, and physicians.

For future DDM fissure research, we recommend adopting a test–intervention–retest methodology. This would require a baseline MRI (test), spinal loading via repeated movements (intervention), and a post-intervention MRI (retest). While some researchers have used this design, it has not been applied to a group with identified radial fissures in the AF (Abdollah et al., 2018).

A considerable challenge in the future will consist in managing the duration of multiparametric MRI sequences, considering both patient comfort and equipment availability. To address this, advanced computational techniques—such as finite element analysis and musculoskeletal multibody modeling—can be leveraged. For instance, Remus and colleagues proposed and validated an open-source model based on the ArtiSynth framework, integrating finite element and multibody approaches to create a comprehensive numerical model of the spine (Remus et al., 2021). This model constitutes a solid foundation for the development of more refined and patient-specific simulations. When combined with deep learning algorithms, such modeling techniques offer promising avenues for characterizing the mechanical properties of the nucleus pulposus (NP) under various loading configurations and for tailoring simulations to individual patients using the concept of numerical twins (Asad et al., 2023). Digital twin approaches have already found applications in clinical practice, notably for simulating postoperative risks following knee surgery (Aubert et al., 2021), as well as for modeling vertebroplasty procedures to predict the mechanical response of the treated vertebral body (Ahmadian et al., 2022a; Ahmadian et al., 2022b). Unlike static image-based assessments, numerical models provide a dynamic and integrative mechanical evaluation, enabling the analysis of displacements and deformations across all three anatomical planes and within the full spinal structure.

### Limitations

The present study has several acknowledged limitations. The high heterogeneity in the proportional meta-analysis influenced by the non-standardization of measurement protocols provided limited evidence to draw objective conclusions. The paucity and heterogeneity of available literature prevented meta-analyses addressing several secondary aims related to the dose-response relationship between bending load and mechanical transformation of the NP and the association between NP migration into a radial fissure and the centralization phenomenon.

No study meeting our inclusion criteria to address association between centralization phenomenon and NP mechanical behavior could be identified. Three studies have demonstrated the high specificity (from 0.7 to 0.95) of centralization phenomenon for discogenic pain (Donelson et al., 1997; Young et al., 2003; Laslett et al., 2005). Although these studies do not demonstrate the association between the behavior of the NP and pain variation, they indicate that the intervertebral disc is the implicated anatomical source of nociception. Therefore, it seems essential to test various hypotheses that could explain this pain behavior. Additionally, we identified a case study showing the association between symptom variation and changes in the position of the NP (identified by MRI and pixel intensity profilometry) (Takasaki et al., 2010). Although this article does not allow for any definitive conclusions, it may be considered proof-of-concept to be replicated on a larger scale. Future research should be conducted to evaluate the relationship between centralization and NP mechanical behavior.

## Conclusion

Based on different methods of image acquisition and analysis to characterize the NP behavior, the DDM was accurate for intact discs in 85.4% of the observations, regardless of the movement plane and the spinal region. Additionally, the DDM was found to be accurate in 81.2% of cases in the sagittal plane for the cervical region and 83.2% for the lumbar region. Accuracy was 90.4% for the lumbar region in frontal plane, respectively. However, based on experimental study results, the relevance of the dynamic disc model (DDM) showed however low evidence quality for intact discs. Whereas data for fissured discs suggested an invalidity of the DDM, no strong conclusion can be reached due to paucity of available data and methodological limitations. Discography and MRI approaches are the most commonly used methods for characterizing NP properties. We recommend exploring new multiparametric MRI approaches in future studies, which may enhance our understanding of disc mechanics as well as inform future clinical assessment and therapeutic pathways.

## Data Availability

The original contributions presented in the study are included in the article/Supplementary Material, further inquiries can be directed to the corresponding author.
