# Supplementary material for: Dynamic behavior of the nucleus pulposus within the intervertebral disc loading: a systematic review and meta-analysis exploring the concept of dynamic disc model
Source: Front Bioeng Biotechnol. 2025 Jun 6;13:1582438. doi: 10.3389/fbioe.2025.1582438 (PMC12179537; doi:10.3389/fbioe.2025.1582438)
Supplement: Supplementary file 1 [file Table1.docx]

Supplementary Material

**Supplemental Digital Content section 1**

**Embase**

'intervertebral disc*':ti,ab AND (degenerat*:ab,ti OR displac*:ab,ti) AND nucle*:ti,ab AND ([english]/lim OR [french]/lim OR [portuguese]/lim OR [spanish]/lim) AND [humans]/lim AND [embase]/lim

**PUBMED**

((intervertebral disc[Title/Abstract]) AND (degenerat*[Title/Abstract] OR displac*[Title/Abstract]) AND (nucle*[Title/Abstract])

**SCIENCE DIRECT**

(intervertebral disc) AND (degenerative OR degeneration OR degenerescence OR displacement) AND (nucleus OR nuclei)

**Google Scholar**

allintitle: nucleus degenerative OR degeneration OR degenerescence OR displacement "intervertebral disc"

**HAL**

nucleus AND (degenerative OR degeneration OR degenerescence OR displacement) AND "intervertebral disc"

**Cochrane Library**

"intervertebral disc" in Title Abstract Keyword AND degenerative OR degeneration OR degenerescence OR displacement in Title Abstract Keyword AND nucleus in Title Abstract Keyword

**CINHAL**

(MH "Intervertebral Disk" OR TI (intervertebral disc* OR intervertebral disk*) OR AB (intervertebral disc* OR intervertebral disk*)) AND (TI (nucle*) OR AB (nucle*)) AND (MH "Intervertebral Disk Displacement" OR TI (degenerat* OR displac*) OR AB (degenerat* OR displac*))

**Supplemental Digital Content section 2**

| **Authors and year** | **URL** | **Reviewer 1 decision** | **Reviewer 2 decision** | **Reviewer 3 decision**  **(arbitration)** | **Shared decision between reviewer 1, 2 and 3** |
| --- | --- | --- | --- | --- | --- |
| Iatridis *et al*., 1996 | https://www.proquest.com/openview/e76160d33a4ee743523132af612967db/1?pq-origsite=gscholar&cbl=18750&diss=y | No measure of nucleus length displacement or deformation according to bending load | Biology |  |  |
| Khoo *et al*.,1995 | <https://www.sciencedirect.com/science/article/pii/135045339590374K> | Modelization study, no direct measurement of nucelar mechanical transformation | Not include in first step |  |  |
| Guo *et al*., 2006 | <https://www.sciencedirect.com/science/article/pii/S0022509606000536> | Modelization study, no evidence of human subject includes, no report of direct measurement of NP mechanical transformation | Not include in first step |  |  |
| Schultz *et al*.,1973 | <https://www.sciencedirect.com/science/article/pii/0021929073900973> | Modelization study, no report of NP mechanical transformation according to bending load | Not include in first step |  |  |
| Holmes *et al*., 1996 | <https://www.sciencedirect.com/science/article/pii/135045339500047X> | Axial compression, no bending load, no report of NP mechanical transformation | no observation of nucleus movement |  |  |
| Fujita *et al*., 2000 | <https://www.sciencedirect.com/science/article/pii/S1350453300000539> | No entire disc, piece of disc mechanically tests, no report of nuclear mechanical transformation | Not include in first step |  |  |
| O’Connell *et al*., 2011 | <https://www.sciencedirect.com/science/article/pii/S1751616111000245> | No bending load, axial compression only, no report of nuclear mechanical transformation | no observation of nucleus movement |  |  |
| Koeller *et al*., 1986 | <https://www.sciencedirect.com/science/article/pii/0021929086901314> | No measure of nucleus length displacement or deformation according to bending load | no observation of nucleus movement |  |  |
| Newell *et a., 2017* | <https://www.sciencedirect.com/science/article/pii/S1751616117300449> | Litterature review | Litterature review |  |  |
| Araujo *et al*., 2012 | <https://www.sciencedirect.com/science/article/pii/S0021929012706218> | Porcine specimen | Animal model |  |  |
| Stokes *et al*., 1986 | <https://www.sciencedirect.com/science/article/pii/0021929086900345> | No direct measurement of the NP mechanical transformation | no observation of nucleus movement |  |  |
| Pham *et al*., 2018 | <https://www.sciencedirect.com/science/article/pii/S002192901730667X> | No measure of nucleus length displacement or deformation according to bending load | no observation of nucleus movement |  |  |
| Iatridis *et al*., 1997 | <https://www.sciencedirect.com/science/article/pii/S0021929097000699> | no measure of the DDM | Not include in first step |  |  |
| Soni *et al*., 1986 | <https://www.sciencedirect.com/science/article/pii/0021929086900321> | No measure of nucleus length displacement or deformation according to bending load | no observation of nucleus movement |  |  |
| Kuroki *et al*., 2004 | <http://dx.doi.org/10.1097/01.brs.0000106683.84600.e5> | Porcine specimen | Not include in first step |  |  |
| O’Connell *et al*., 2010 | <http://dx.doi.org/10.1002/jor.21232> | very interesting study, data collected should have permitted to the authors to do calculation in which we are interested in. However, they mainly focus onto AF behavior and very low descriptions of methods and results are provide for the NP | Not include in first step |  |  |
| Wilson *et al*., 2021 | <http://dx.doi.org/10.1038/s41598-020-77577-y> | very interesting paper, with very good methods which could have been include. However, authors did not differentiate NP mechanics to AF mechanics. They assess the whole disc transformation according to bending load, not the NP transformation | no measure of the DDM |  |  |
| Fennell *et al*., 1996 | <http://dx.doi.org/10.1097/00007632-199612010-00009> | Include | Include |  |  |
| Edmonston *et al*., 2000 | <http://dx.doi.org/10.1054/math.2000.0356> | Include | Include |  |  |
| Abdollah *et al*., 2017 | <http://dx.doi.org/10.1016/j.msksp.2017.11.008> | Uncertain | Include | Uncertain | No screening for radial fissure |
| Takasaki *et al*., 2010 | <http://dx.doi.org/10.1179/106698110X12640740712455> | Include | Include |  |  |
| Edwards *et al*., 2001 | <http://dx.doi.org/10.1097/00007632-200108150-00005> | No measure of nucleus length displacement or deformation according to bending load | Not include in first step |  |  |
| Nazari *et al*., 2011 | <http://dx.doi.org/10.1016/j.clinbiomech.2011.09.011> | Include | Include |  |  |
| Yang *et al*., 2019 | <http://dx.doi.org/10.1115/1.4043029> | Modelization study | Include |  |  |
| Alexander *et al*., 2007 | <http://dx.doi.org/10.1097/BRS.0b013e318067dccb> | Include | Include |  |  |
| Brulliard, 2014 | <https://hal.archives-ouvertes.fr/tel-01224563> | Modelization study | Model |  |  |
| Kim *et al*., 2017 | <https://search.ebscohost.com/login.aspx?direct=true&db=rzh&AN=122646485&site=ehost-live> | Include | Include |  |  |
| Adams *et al*., 2000 | <https://www.embase.com/search/results?subaction=viewrecord&id=L30115560&from=export> | No measure of NP mechanical transformation | no observation of nucleus movement |  |  |
| Chung *et al*., 2013 | <https://www.embase.com/search/results?subaction=viewrecord&id=L71205701&from=export> | Same report than Kim et al., which are already include, here we have a conference paper, we prefer to keep the full paper include | Duplicate from Kim et al |  |  |
| Van Heeswijk *et al*., 2017 | <https://search.ebscohost.com/login.aspx?direct=true&db=rzh&AN=125717107&site=ehost-live> | Ovine specimen | Not include in first step |  |  |
| Brault *et al*., 1997 | <https://search.ebscohost.com/login.aspx?direct=true&db=rzh&AN=138084602&site=ehost-live> | Include | Include |  |  |
| Kolber and Hanney, 2009 | <https://search.ebscohost.com/login.aspx?direct=true&db=rzh&AN=105403248&site=ehost-live> | Litterature review |  |  |  |
| Laws *et al*., 2016 | <https://search.ebscohost.com/login.aspx?direct=true&db=rzh&AN=118091825&site=ehost-live> | no measure of the DDM | Not include in first step |  |  |
| Szulc *et al*., 2012 | <https://www.cochranelibrary.com/central/doi/10.1002/central/CN-01017726/full> | no measure of the DDM |  |  |  |
| Spivak *et al*., 2015 | <https://www.cochranelibrary.com/central/doi/10.1002/central/CN-01655003/full> | no measure of the DDM | no observation of nucleus movement |  |  |
| Prybyla *et al*., 2006 | <https://www.embase.com/search/results?subaction=viewrecord&id=L44691996&from=export> | No measure of NP mechanical transformation | Not include in first step |  |  |
| Muriuki *et al*., 2014 | <https://www.embase.com/search/results?subaction=viewrecord&id=L71675777&from=export> | no measure of the DDM | Imagery only |  |  |
| Goobar *et al*., 1988 | <https://www.embase.com/search/results?subaction=viewrecord&id=L18030489&from=export> | no measure of the DDM | no observation of nucleus movement |  |  |
| Deneuville *et al*., 2020 | <https://www.embase.com/search/results?subaction=viewrecord&id=L634127870&from=export> | Ovine specimen | Not include in first step |  |  |
| Ranu *et al*., 1985 | <https://www.embase.com/search/results?subaction=viewrecord&id=L15085839&from=export> | no measure of the DDM | no observation of nucleus movement |  |  |
| Fazey *et al*., 2006 | https://doi.org/10.1016/j.clinbiomech.2005.12.008 | Include | Include |  |  |
| Gill *et al*., 1987 | https://doi.org/10.1016/0268-0033(87)90083-0 | Include | Include |  |  |
| Perea *et al*., 2007 | https://doi.org/10.1002/mrm.21231 | Not include in first step | Imagery only |  |  |
| Sloan *et al*., 2020 | https://doi.org/10.1126/scitranslmed.aay2380 | Not include in first step | Surgery |  |  |
| Culbert *et al*., 2022 | http://dx.doi.org/10.1039/D1BM01589C |  | surgery |  |  |
| Shirazi-Adl *et al*., 2020 | <https://www.sciencedirect.com/science/article/pii/S0021929020300348> | Not include in first step | no observation of nucleus movement |  |  |
| Chaffin, 1969 | <https://www.sciencedirect.com/science/article/pii/0021929069900189> | Not include in first step | Model |  |  |
| Panagiotacopoulos *et al*., 1987 | <https://www.sciencedirect.com/science/article/pii/0021929087901448> | Not include in first step | Education |  |  |
| Schnebel *et al*., 1998 | https://doi.org/10.1097/00007632-198803000-00014 | Include | Include |  |  |
| Tamoud *et al*., 2021 | <https://www.sciencedirect.com/science/article/pii/S0020740321002939> | Model only | Include | Model without experimental comparison |  |
| Benboukha, 2011 | <https://www.sciencedirect.com/science/article/pii/S1876610211014585> | Not include in first step | Model |  |  |
| Gonzalez *et al*., 2008 | <https://www.sciencedirect.com/science/article/pii/S0021929008702550> | Not include in first step | Imagery only |  |  |
| Pollitine *et al*., 2006 | <https://www.sciencedirect.com/science/article/pii/S0021929006829817> | Not include in first step | no observation of nucleus movement |  |  |
| Périé et al., 2001 | https://doi.org/10.1016/s0730-725x(01)00452-0 | Include | No bending movement | Include |  |
| Panjabi *et al*., 1975 | <https://www.sciencedirect.com/science/article/pii/0021929075900858> | Not include in first step | no observation of nucleus movement |  |  |
| Malandrino *et al*., 2018 | <https://www.sciencedirect.com/science/article/pii/B9780128128510000069> | Not include in first step | education |  |  |
| Galbusera *et al*., 2022 | <https://www.sciencedirect.com/science/article/pii/B9780128244814000287> | Not include in first step | education |  |  |
| Yang *et al*., 1988 | <https://www.sciencedirect.com/science/article/pii/0021929088900590> | Not include in first step | no observation of nucleus movement |  |  |
| Lanza *et al*., 2020 | <https://www.sciencedirect.com/science/article/pii/B9780128184226000915> | Not include in first step | biology |  |  |
| Takavoli *et al*., 2020 | <https://www.sciencedirect.com/science/article/pii/S1742706120303299> | Not include in first step | no observation of nucleus movement |  |  |
| Holm *et al*., 2006 | <https://www.sciencedirect.com/science/article/pii/S0021929006849614> | Not include in first step | no observation of nucleus movement |  |  |
| Adams *et al*., 1998 | <https://www.sciencedirect.com/science/article/pii/S002192909880208X> | Measure of stress, not NP deformation | Include | Conference abstract, no measure of NP displacement exclude |  |
| Spilker *et al*., 1981 | <https://www.sciencedirect.com/science/article/pii/0021929081901329> | Not include in first step | no observation of nucleus movement |  |  |
| Ogon *et al*., 2020 | <https://www.sciencedirect.com/science/article/pii/S0021929008002339> | Not include in first step | no observation of nucleus movement |  |  |
| O’Connell *et al*., 2010 | <http://dx.doi.org/10.1016/j.xnsj.2020.100044> | Not include in first step | Imagery only |  |  |
| Krag *et al*., 1987 | <http://dx.doi.org/10.1097/00007632-198712000-00011> | No direct observation of the NP, marker are visible not the NP | Include | No direct observation of the NP exclude |  |
| Van Risjbergen *et al*., 2016 | <http://dx.doi.org/10.1007/s10237-016-0835-9> | Not include in first step | Model |  |  |
| Kramer *et al*., 1993 | <https://www.ncbi.nlm.nih.gov/pubmed/8234669> | Not include in first step | Imagery only |  |  |
| Schütz *et al*., 2019 | <http://dx.doi.org/10.1007/s00132-018-03672-8> | Not include in first step | Imagery only |  |  |
| Rodriguez *et al*., 2011 | <http://dx.doi.org/10.1002/jor.21513> | Not include in first step | Imagery only |  |  |
| Kumaresan *et al*., 2000 | <https://www.ncbi.nlm.nih.gov/pubmed/10834223> | Not include in first step | Imagery only |  |  |
| Walter *et al*., 2017 | <http://dx.doi.org/10.1148/radiol.2017162287> | Not include in first step | biology |  |  |
| Jenkins *et al*., 1985 | <http://dx.doi.org/10.1259/0007-1285-58-692-705> | Not include in first step | Imagery only |  |  |
| DeLucca *et al*., 2016 | <http://dx.doi.org/10.1002/jor.23315> | Not include in first step | Imagery only |  |  |
| Xiong *et al*., 2018 | <https://www.ncbi.nlm.nih.gov/pubmed/29511438> | Not include in first step | Imagery only |  |  |
| Tsantrizos et al., 2005 | https://doi.org/10.1097/01.brs.0000181052.56604.30 | Uncertain | Include | Uncertain | Exclude : nodirect observation of NP, marker instead |
| Wang *et al*., 2017 | <http://dx.doi.org/10.3892/etm.2017.4786> | Not include in first step | Imagery only |  |  |
| Foltz *et al*., 2017 | <http://dx.doi.org/10.1115/1.4037549> | Not include in first step | Imagery only |  |  |
| Fredericson *et al*., 2001 | https://doi.org/10.1016/s1529-9430(01)00014-6 | Annular deformation not NP | no observation of nucleus movement |  |  |
| Fontes *et al*., 2019 | <http://dx.doi.org/10.1371/journal.pone.0218121> | Not include in first step | Imagery only |  |  |
| Begg, 1954 | <http://dx.doi.org/10.1302/0301-620X.36B2.180> | Not include in first step | Imagery only |  |  |
| Cincu *et al*., 2015 | <http://dx.doi.org/10.4103/1793-5482.151504> | Not include in first step | Surgery |  |  |
| Chen *et al*., 2014 | <http://dx.doi.org/10.1371/journal.pone.0087856> | Not include in first step | Imagery only |  |  |
| Pachowsky *et al*., 2018 | <http://dx.doi.org/10.1177/1947603518758434> | Not include in first step | Surgery |  |  |
| Ellingson *et al*., 2014 | <http://dx.doi.org/10.1002/jor.22633> | Not include in first step | Imagery only |  |  |
| Dolan and Adams, 2001 | <http://dx.doi.org/10.1016/s0268-0033(00)00096-6> | Not include in first step | model |  |  |
| Yang *et al*., 2021 | <http://dx.doi.org/10.1002/ca.23816> | Not include in first step | Imagery only |  |  |
| Yang *et al*., 2019 | <http://dx.doi.org/10.1115/1.4043029> | Model Only | include | Model without comparison to experimental data |  |
| Fazey *et al*., 2010 | https://doi.org/10.1007/s00586-010-1339-4 | Include | Include |  |  |
| Ogon *et al*., 2020 | <http://dx.doi.org/10.31616/asj.2019.0231> | Not include in first step | Imagery only |  |  |
| Li *et al*., 2016 | <https://www.ncbi.nlm.nih.gov/pubmed/26876403> | Not include in first step | Surgery |  |  |
| Galley *et al*., 2018 | <http://dx.doi.org/10.5334/jbsr.1501> | Not include in first step | Imagery only |  |  |
| Nagashima *et al*., 2013 | <http://dx.doi.org/10.1177/0363546513495173> | Not include in first step | Imagery only |  |  |
| Liu *et al*., 2021 | <http://dx.doi.org/10.1177/1947603521996793> | Not include in first step | Imagery only |  |  |
| Rahyussalim *et al*., 2019 | <http://dx.doi.org/10.31616/asj.2019.0046> | Not include in first step | Imagery only |  |  |
| Zhang *et al*., 2021 | <http://dx.doi.org/10.1155/2021/2869488> | Not include in first step | model |  |  |
| Beattie *et al*., 1994 | https://doi.org/10.1097/00007632-199409150-00017 | Include | Include |  |  |
| El maazi et al., 2019 | https://doi.org/10.1016/j.msksp.2019.07.002 | Include | Include |  |  |
| Beekmans *et al*., 2018 | <http://dx.doi.org/10.1002/jsp2.1005> | Not include in first step | no observation of nucleus movement |  |  |
| Gay *et al*., 2007 | <http://dx.doi.org/10.1016/j.spinee.2007.07.398> | Not include in first step | no observation of nucleus movement |  |  |
| Yoon *et al*., 2016 | <http://dx.doi.org/10.1016/j.mri.2016.04.024> | Not include in first step | Imagery only |  |  |
| Meakin *et al*., 2001 | <http://dx.doi.org/10.1016/s0268-0033(00)00075-9> | Not include in first step | Surgery |  |  |
| Adams *et al*., 1982 | <https://www.embase.com/search/results?subaction=viewrecord&id=L13087410&from=export> | Not include in first step | Imagery only |  |  |
| Moore *et al*., 1996 | <http://dx.doi.org/10.1097/00007632-199609150-00018> | Not include in first step | Imagery only |  |  |
| Schmidt *et al*., 2007 | <http://dx.doi.org/10.1016/j.clinbiomech.2007.07.008> | Not include in first step | model |  |  |
| Xiu *et al*., 2012 | <http://dx.doi.org/10.3233/XST-2012-0336> | Not include in first step | Surgery |  |  |
| Seroussi *et al.*, 1989 | https://doi.org/10.1002/jor.1100070117 | No direct observation of the NP | Include | No direct of the observation exclude |  |
| Fogwe *et al*., 2022 | <https://www.ncbi.nlm.nih.gov/pubmed/29262010> | Not include in first step | clinique |  |  |
| Takasaki, 2015 | https://doi.org/10.1179/2042618613y.0000000059 | Include | Include |  |  |
| Zhu *et al*., 2015 | <http://dx.doi.org/10.1186/s13018-014-0142-z> | Not include in first step | Surgery |  |  |
| Detiger *et al*., 2015 | <http://dx.doi.org/10.1177/0885328215611946> | Not include in first step | Surgery |  |  |
| Wu *et al*., 2018 | <http://dx.doi.org/10.3969/j.issn.1003-0034.2018.08.007> | Not include in first step | Surgery |  |  |
| Sharifi *et al*., 2014 | <http://dx.doi.org/10.1002/term.1866> | Not include in first step | Surgery |  |  |
| Ma *et al*., 2014 | <https://www.embase.com/search/results?subaction=viewrecord&id=L373859814&from=export> | Not include in first step | Imagery only |  |  |
| Wang *et al*., 2015 | <https://www.embase.com/search/results?subaction=viewrecord&id=L614586214&from=export> | Not include in first step | Imagery only |  |  |
| Fazey *et al*., 2013 | https://doi.org/10.1016/j.clinbiomech.2013.03.009 | Include | Include |  |  |
| Lebkowski, 2002 | <https://www.embase.com/search/results?subaction=viewrecord&id=L35108172&from=export> | Not include in first step | Imagery only |  |  |
| Lipscomb *et al*., 2014 | <https://www.embase.com/search/results?subaction=viewrecord&id=L52997858&from=export> | Not include in first step | model |  |  |
| Cai *et al*., 2015 | <https://www.embase.com/search/results?subaction=viewrecord&id=L612132372&from=export> | Not include in first step | no observation of nucleus movement |  |  |
| Kanamori *et al*., 2013 | <https://www.embase.com/search/results?subaction=viewrecord&id=L369871132&from=export> | Not include in first step | Imagery only |  |  |
| Parent *et al.* | https://doi.org/10.1097/01.brs.0000245834.30646.aa | Annular deformation not NP | no observation of nucleus movement |  |  |
| Bimey *et al*., 1992 | <https://www.cochranelibrary.com/central/doi/10.1002/central/CN-00090429/full> | Not include in first step | Imagery only |  |  |
| Ma *et al*., 2021 | <https://www.cochranelibrary.com/central/doi/10.1002/central/CN-02302161/full> | Not include in first step | clinique |  |  |
| Ellingston *et al*., 2012 | <https://www.embase.com/search/results?subaction=viewrecord&id=L70895789&from=export> | Exclude for 1rst step | Include | Conference abstract which not report displacement measurement of the NP |  |
| Zhang *et al*., 2011 | <https://www.embase.com/search/results?subaction=viewrecord&id=L364737324&from=export> | Not include in first step | Imagery only |  |  |
| Chu *et al*., 2008 | <https://www.embase.com/search/results?subaction=viewrecord&id=L351728412&from=export> | Not include in first step | Imagery only |  |  |
| Vadalá *et al*., 2012 | <https://www.embase.com/search/results?subaction=viewrecord&id=L71314465&from=export> | Not include in first step | Imagery only |  |  |
| Willis *et al*., 2007 | <https://www.embase.com/search/results?subaction=viewrecord&id=L46891262&from=export> | Not include in first step | education |  |  |
| Hall *et al*., 2000 | <https://www.embase.com/search/results?subaction=viewrecord&id=L30740116&from=export> | Not include in first step | education |  |  |
| Adams, 2009 | <https://www.embase.com/search/results?subaction=viewrecord&id=L354810564&from=export> | Not include in first step | education |  |  |
| Tertti *et al*., 1991 | <https://www.embase.com/search/results?subaction=viewrecord&id=L21205691&from=export> | Not include in first step | Imagery only |  |  |
| Gangi, 2010 | <https://www.embase.com/search/results?subaction=viewrecord&id=L70288779&from=export> | Not include in first step | education |  |  |
| Kallewaard *et al*., 2014 | <https://www.embase.com/search/results?subaction=viewrecord&id=L71512390&from=export> | Not include in first step | education |  |  |
| Weishaupt et al., 2000 | https://doi.org/10.1148/radiology.215.1.r00ap06247 | Annular deformation not NP | no observation of nucleus movement |  |  |
| Mekail *et al*., 2000 | <https://www.embase.com/search/results?subaction=viewrecord&id=L30683665&from=export> | Not include in first step | Imagery only |  |  |
| Karakida *et al*., 2003 | <https://www.embase.com/search/results?subaction=viewrecord&id=L36546727&from=export> | Not include in first step | Imagery only |  |  |
| Frobin *et al*., 2001 | <https://www.cochranelibrary.com/central/doi/10.1002/central/CN-00327320/full> | Not include in first step | Imagery only |  |  |
| Vining *et al*., 2014 | <https://www.cochranelibrary.com/central/doi/10.1002/central/CN-01077452/full> | Not include in first step | Imagery only |  |  |
| El Barzouhi *et al*., 2016 | <https://www.cochranelibrary.com/central/doi/10.1002/central/CN-01260056/full> | Not include in first step | Imagery only |  |  |
| Zamani *et al*., 1998 | https://doi.org/10.1002/jmri.1880080622 | Annular deformation not NP | no observation of nucleus movement |  |  |
| Boos *et al*., 1993 | <https://www.cochranelibrary.com/central/doi/10.1002/central/CN-00094381/full> | Not include in first step | Imagery only |  |  |
| Zou *et al*., 2009 | https://doi.org/10.1097/brs.0b013e3181b32998 | Annular deformation not NP | no observation of nucleus movement |  |  |
| Miyazaki *et al*., 2008 | <https://www.cochranelibrary.com/central/doi/10.1002/central/CN-00640120/full> | Not include in first step | Imagery only |  |  |
| Tan *et al*., 2000 | <https://www.cochranelibrary.com/central/doi/10.1002/central/CN-01776791/full> | Not include in first step | Imagery only |  |  |
| Berg *et al*., 2012 | <https://www.cochranelibrary.com/central/doi/10.1002/central/CN-00970148/full> | Not include in first step | Imagery only |  |  |
| Apfel *et al*., 2010 | <https://www.cochranelibrary.com/central/doi/10.1002/central/CN-00767818/full> | Not include in first step | Surgery |  |  |
| Chen *et al*., 2014 | <https://www.embase.com/search/results?subaction=viewrecord&id=L373859802&from=export> | Not include in first step | Surgery |  |  |
| Goto *et al*., 2002 | <https://www.embase.com/search/results?subaction=viewrecord&id=L34456645&from=export> | Not include in first step | model |  |  |
| Hasegawa *et al*., 1991 | <https://www.embase.com/search/results?subaction=viewrecord&id=L22058357&from=export> | Not include in first step | no observation of nucleus movement |  |  |
| Shah et al., 1978 | The distribution of surface strain in the cadaveric lumbar spine. | Uncertain | Include | Uncertain | Include |
| Papakonstantinou, 2011 | <https://www.embase.com/search/results?subaction=viewrecord&id=L70624061&from=export> | Not include in first step | education |  |  |
| Zou *et al*., 2006 | <https://www.embase.com/search/results?subaction=viewrecord&id=L46566606&from=export> | Not include in first step | model |  |  |
